# A Pathway for Sugar Production from Agricultural Waste Catalyzed by Sulfonated Magnetic Carbon Microspheres

**DOI:** 10.3390/molecules30132675

**Published:** 2025-06-20

**Authors:** Maoru Xu, Yanfeng Duan, Hongfu Li, Shoulin He, Xingyu Zi, Yanting Zhao, Cheng Jiao, Xiaoyun Li

**Affiliations:** 1 Dali Tobacco Bureau of Yunnan Province, Dali 671000, China; xmaoru1856@163.com (M.X.); zy_huang2022@163.com (H.L.); he0325@163.com (S.H.); gkysciaccept@163.com (X.Z.); 18987237598@163.com (Y.Z.); 2 School of Agriculture and Biotechnology, Sun Yat-sen University, Shenzhen 518107, China; duanyf7@mail2.sysu.edu.cn

**Keywords:** acid catalysis, biomass, effective separation, environmentally friendly, magnetic carbon materials, reducing sugar

## Abstract

Lignocellulose is an important renewable biomass resource. However, at present, there is a lack of efficient and environmentally friendly catalytic systems that can selectively convert lignocellulose components into high-value sugars, and the value realization of agricultural waste (such as straw) remains challenging. Carbon-based solid acids are used in the valorization of biomass due to their simple preparation and excellent catalytic performance. In this study, the magnetic carbon microspheres catalyst was prepared using concentrated sulfuric acid and hydroxyethyl sulfonic acid as sulfonating agents. Two sulfonation catalysts were applied to the hydrolysis of typical agricultural waste (rice straw). The performance of catalyst conversion to reducing sugar was compared, and the glucose yield was lower than 30%. The sulfonation catalyst of hydroxyethyl sulfonic acid obtained a higher yield of pentose (76.67%) than that of concentrated sulfuric acid (74.25%) in 110 min. The optimal reaction conditions were found: substrate was 0.04 g straw, catalyst was 0.04 g, H_2_O/γ-valerolactone ratio was 8:2 in the solvent, and the reaction time was 110 min at 140 °C. Under these conditions, the sulfonation properties of hydroxyethyl sulfonic acid as a green sulfonating agent are similar to those of concentrated sulfuric acid. Its excellent catalytic performance is attributed to the medium B/L acid density ratio on the catalyst surface. In addition, the prepared catalyst can be effectively separated from the reaction residue in the catalytic system. This work provides a green catalytic system for the high-value utilization of agricultural waste from renewable carbon sources.

## 1. Introduction

With the development of society, resource issues have emerged as a major topic that humanity needs to address urgently. For the increasing demands of human life, the non-renewable resources of traditional fossil fuels will gradually be depleted [1]. Moreover, burning fossil fuels emits large amounts of carbon dioxide, other greenhouse gases, and particulate matter [2,3]. This makes the greenhouse effects worse. Consequently, an increasing number of new renewable energy sources are being developed and utilized [4,5]. Among them, biomass energy stands out as the energy stored in biomass, primarily obtained through the process of photosynthesis in plants. Biomass, serving as an energy carrier, mainly exists in the form of organic carbon found in vegetation, aquatic plants, agricultural residues, and organic waste [6]. It is an important source for producing biomass-based fuel and chemicals [7,8]. Approximately 170 billion tons of biomass resources are generated globally each year, but only 3% is used as energy, accounting for 14% of the world’s total energy consumption [9]. Therefore, improving the utilization efficiency of biomass resources can greatly meet human energy needs and mitigate the impact of fossil fuel shortages on humanity.

Lignocellulosic biomass is the most abundant and renewable biomass resource [10,11]. It is crucial for replacing traditional methods in fuel and basic chemical production. Lignocellulosic biomass is widely found in agricultural residues such as corn stover, bagasse, rice straw, and corn cobs. Additionally, there is a large amount of lignocellulosic biomass in forestry by-products such as pine wood and sawdust [12,13]. These materials are rich in organic matter and various mineral elements. Lignocellulosic biomass is primarily composed of cellulose, hemicellulose, and lignin. Each substance can be utilized differently to produce fuels or chemicals such as ethanol and furfural [14,15,16]. Differences in their chemical composition and structure lead to variations in their reactivity. The predominant component, cellulose, can be hydrolyzed to produce glucose, which can then be fermented by yeast to produce ethanol. Additionally, dehydration of glucose can yield platform chemicals such as 5-hydroxymethylfurfural and furfural [17]. It is estimated that nearly half of the organic carbon in the biosphere exists in the form of cellulose [18,19]. Hemicellulose can be hydrolyzed to produce xylose and arabinose, which can be further processed to produce xylitol [20]. Structurally, cellulose forms microfibrils that constitute the basic framework of plant cell walls with hemicellulose acting as an adhesive and lignin as a filler. The different binding forces among these molecules result in a highly recalcitrant structure, making the hydrolysis of lignocellulose particularly challenging [21].

Heterogeneous catalysts are widely used in the conversion of lignocellulosic biomass [22,23]. Carbon-based solid acids garner particular attention because of their simple preparation and excellent performance [24]. The preparation methods of carbon-based solid acid mainly include the carbonization method, sulfonation method, oxidation method, etc. Among them, the sulfonation method uses a sulfonation reagent to react with functional groups such as hydroxyl groups on the surface of carbon-based materials to produce acidic functional groups such as sulfonic acid groups [25]. Compared with similar catalysts, sulfonated carbon materials prepared by the sulfonation method show excellent selectivity in catalytic process, reducing sugar production. They not only have higher efficiency but also cause less damage to the pore structure [26]. Thus, the catalyst is endowed with a larger specific surface area and a higher sulfonic acid group density, which improves the performance of the catalyst. This material has a chemical function of high stability and selectivity under optimal conditions [27]. Bai et al. successfully prepared several carbon-based solid acids from various biomass sources (glucose, microcrystalline cellulose, bamboo, and rice husk). These unique acids were then employed as catalysts to catalyze cellulose hydrolysis. Using a ball-milling method and dissolving cellulose in ionic liquids, they achieved a maximum total reducing sugar yield of 81.8% [28]. Through the sulfonation of a carbon catalyst, the catalytic effect of biomass can be significantly improved. Weerasai et al. synthesized solid sulfonated carbon-based catalysts from three carbon precursors (sucrose, glucose, and xylose). Alkaline oxidation pretreatment of biomass increased the reducing sugar yield (up to 40.7%) under specific conditions. Besides biomass pretreatment, sulfonated carbon-based solid acids can further enhance lignocellulose conversion [29]. Qi et al. synthesized a novel carbon-based solid (C-SO_3_H) acid catalyst using a one-step hydrothermal carbonization method with microcrystalline cellulose and sulfuric acid. Under optimal pretreatment conditions (140 °C, 6 h, 0.25 g corn cobs, 0.25 g catalyst, and 25 mL H_2_O), they achieved a xylose yield of 78.1% [30]. Xu et al. prepared catalyst materials by a carbonized and sulfonated mixture of glucose and p-toluene sulfonic acid and enhanced the acidity with 30% H_2_O_2_ oxidation. The catalyst resulted in a xylose yield of 78% from corn cobs hydrolysis [31]. Sulfonated carbon-based solid acids exhibit high catalytic activity in the catalytic production of sugar from biomass [32,33]. However, using carbon-based solid acids as catalysts in hydrolysis systems presents a significant challenge: the direct separation of the catalyst from the solid residues produced after lignocellulosic biomass hydrolysis.

In this study, the sulfonated magnetic carbon microsphere materials were prepared by two steps. The resulting sulfonated magnetic carbon microsphere materials were applied to directly catalyze the hydrolysis of rice straw to reducing sugars, and the yields of glucose and pentose were 22.23% and 76.67%, respectively. Compared with previous studies, hydroxyethyl sulfonic acid was selected as the sulfonating agent in this study, which has higher catalytic activity and selectivity. During the sulfonation process, the generation and emission of harmful substances can be reduced, significantly lowering the discharge of pollutants. Meanwhile, the unique magnetic property enhances the recovery efficiency of the catalyst. Therefore, the magnetic sulfonation catalyst prepared in this study has made remarkable progress in solving the catalyst separation problem in the current hydrolysis system. It also provides new ideas and methods for sustainable utilization in the field of biomass transformation.

## 2. Results and Discussion

### 2.1. Characterization of a Carbon-Based Solid Acid Catalyst

**Figure 1:**
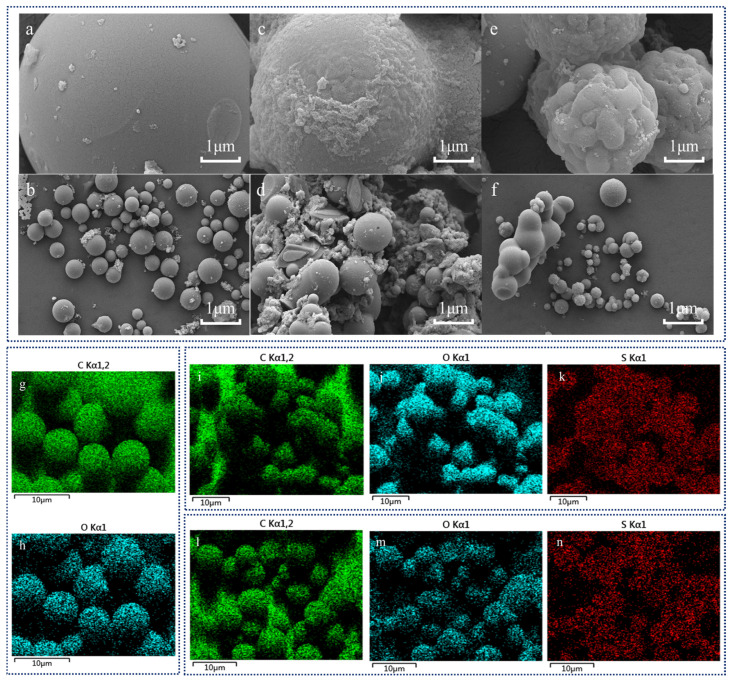
The SEM image of the magnetic carbon materials before and after sulfonation: (**a**,**b**) before sulfonation, (**c**,**d**) sulfonated with concentrated sulfuric acid, (**e**,**f**) sulfonated with hydroxyethyl sulfonic acid. The EDX image of the magnetic carbon materials before and after sulfonation: (**g**,**h**) before sulfonation, (**i**–**k**) sulfonated with concentrated sulfuric acid, (**l**–**n**) sulfonated with hydroxyethyl sulfonic acid.

The magnetic carbon materials were prepared by hydrothermal treatment. The SEM results of the magnetic carbon materials before and after sulfonation are shown in Figure 1. After sulfonation, most carbon microspheres exhibited aggregation, and their surfaces changed from smooth to a raised state, forming an amorphous structure. This indicated an increase in surface functional groups on the magnetic carbon material post-sulfonation, resulting in a completely non-porous and more stable catalyst structure [34]. The EDX image shows the elemental distribution in the catalyst before and after sulfonation, with the S elements showing a uniform distribution in the material after sulfonation. The sulfonated material had a fine grain, and the phase interface was clearer and the phase distribution was more uniform. The size and uniform distribution of the material particle shape were significantly improved before and after sulfonation. Compared to traditional carbon materials, the magnetic carbon material synthesized in this study exhibited a larger specific surface area (Figure 1). This larger specific surface area can provide more active sites for adsorption of reactants. The magnetic carbon materials treated with two different sulfonating agents (Figure 1c–f) showed some damaged microsphere structures. Through statistical analysis of SEM images, it was found that the magnetic carbon material sulfonated with hydroxyethyl sulfonic acid (average diameter 1.33 μm) exhibited smaller microspheres than that sulfonated with concentrated sulfuric acid (average diameter 1.51 μm). This smaller microsphere morphology may contribute to the diffusion rate of the substrate inside the catalyst, making it easier for the reactants to reach the active site inside the catalyst.

**Figure 2:**
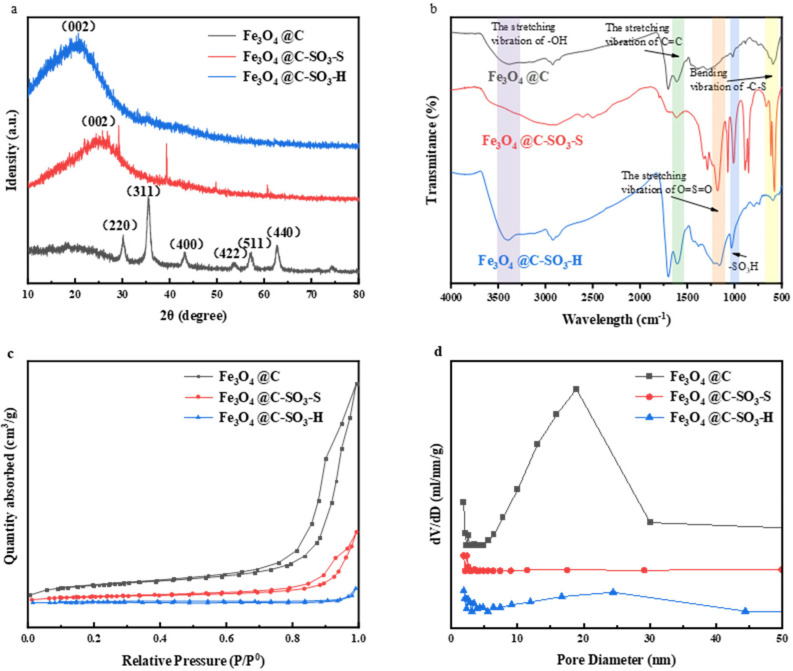
XRD (**a**), infrared spectra (**b**), nitrogen adsorption–desorption isotherms (**c**), and pore diameter distribution (**d**) of Fe_3_O_4_@C, Fe_3_O_4_@C-S, and Fe_3_O_4_@C-H.

The XRD characterization results of the synthesized material before and after sulfonation are shown in Figure 2a. The magnetic carbon material before sulfonation exhibited characteristic diffraction peaks of Fe_3_O_4_ at 2θ values of 30°, 35°, 43°, 57°, and 62° [35,36]. The remaining peaks coincided with the standard peaks of complex carbon-oxygen compounds. After sulfonation, the diffraction peaks of the two catalysts were primarily concentrated in the 10–30° range, all of which were broad peaks corresponding to the characteristic diffraction peak of the C (002) plane. This suggested that the catalysts post-sulfonation exist in the form of amorphous carbon. These findings indicated that the sulfonation reaction not only introduced sulfonic acid groups but also altered the structure of the carbon materials [37]. After sulfonation, the material retained its magnetic properties despite disappearance of the diffraction peaks corresponding to Fe_3_O_4_. This implied that Fe_3_O_4_ serves as the magnetic core, enveloped by the carbon material, and that the surface-attached free Fe_3_O_4_ disappears after sulfonation.

The infrared spectra of the magnetic carbon materials before and after sulfonation are shown in Figure 2b. The results indicated that more functional groups were exposed after sulfonation. The stretching vibration peak around 3400 cm^−1^ corresponded to -OH groups. The peak at 1609–1613 cm^−1^ corresponded to the stretching vibrations of aromatic C=C bonds. The characteristic peak of the sulfonic acid group appeared in the region of 1000–1400 cm^−1^. The peak at 1171 cm^−1^ was attributed to the O=S=O stretching vibrations. This indicates the presence of sulfonic acid groups on the surface of the sample. The peak at 1068 cm^−1^ was characteristic of -SO_3_H groups, and the peak at 610 cm^−1^ corresponded to C-S bending vibrations [38,39]. The C-S bond is the bonding bond between sulfur atoms and carbon atoms in the sulfonic acid group, and its bending vibration further supports the presence of the sulfonic acid group on the sample surface. The characteristic peaks before and after sulfonation of the magnetic carbon materials were compared. It showed that both the magnetic carbon materials sulfonated with concentrated sulfuric acid and those sulfonated with hydroxyethyl sulfonic acid exhibited an increase in the O=S=O functional groups and -SO_3_H functional groups on their surfaces. This indicates that sulfonic acid groups were successfully loaded onto the surface of the carbon microspheres, which could facilitate intermolecular dehydration [40]. The characteristic peak intensity of the sulfonic acid group in the spectrum was uniform, showing a narrow shape, and there was no obvious peak splitting or asymmetry. This indicates that the sulfonic acid group was uniformly dispersed on the surface of the sample.

The specific surface area, pore volume, and pore diameter of the magnetic carbon materials before and after sulfonation are shown in Table S1. The specific surface area of the two magnetic carbon materials after sulfonation (both <4.7 m^2^/g) was significantly smaller than that before sulfonation (13.0379 m^2^/g). This indicates that the surface of the magnetic carbon materials lacks significant pore structures, which was consistent with the SEM characterization [41,42,43]. Figure 2c shows the adsorption-desorption isotherms of the magnetic carbon materials, which is assigned to the characteristics of Type III isotherms [44]. The initial part of the isotherm raised slowly, followed by a sharp increase in adsorption due to capillary condensation in the latter part. However, the adsorption capacity of the magnetic carbon materials did not increase significantly after sulfonation. This phenomenon may be due to the substantial reduction in specific surface area and pore volume, leading to a decreased adsorption capacity. As can be seen from the pore size distribution curve in Figure 2d, the pore sizes of the three magnetic carbon materials were between 2 and 50 nm, corresponding to the mesoporous structure. However, the total pore volume was small, which shows atypical adsorption characteristics of mesoporous materials. The specific surface area and total pore volume of the sulfonated magnetic carbon materials were significantly smaller than those before sulfonation. This proved that sulfonic acid groups were loaded onto the carbon materials, filling the surface pores [45].

**Figure 3:**
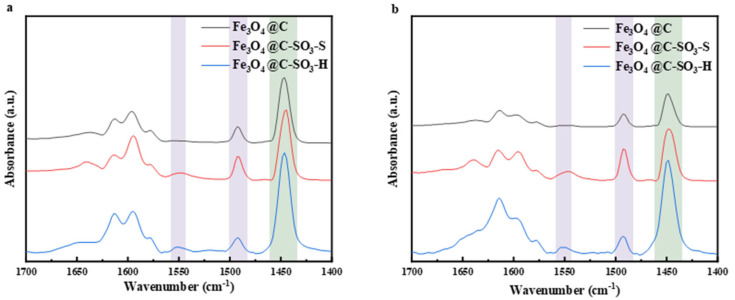
The pyridine infrared spectra of Fe_3_O_4_@C, Fe_3_O_4_@C-S, and Fe_3_O_4_@C-H at different temperatures: (**a**) 150 °C and (**b**) 200 °C.

The pyridine infrared spectra are shown in Figure 3. The characteristic peaks at 1540–1548 cm^−1^ corresponded to the pyridine adsorbed on Bronsted acid (B acid) sites, and the peaks at 1445–1454 cm^−1^ corresponded to the pyridine adsorbed on Lewis acid (L acid) sites. The peak at 1490 cm^−1^ was attributed to the combination of B acid and L acid sites [46,47]. The Fe_3_O_4_@C-S showed a larger peak area at the B acid sites. Fe_3_O_4_@C-H presented a larger peak area at the L acid sites. Additionally, the peak areas corresponding to B acid, L acid, and their combination in both sulfonated magnetic carbon materials were significantly larger than the carbon materials before sulfonation. The results indicated that the magnetic carbon materials have enhanced acid catalytic performance after sulfonation. Table 1 shows the acid site densities of B and L acids in the three carbon materials, as well as the B/L ratio, which is the ratio of the acid site densities of B acid to L-acid. The magnetic carbon materials after sulfonation exhibited significantly higher densities of Brønsted (B), Lewis (L), and total acid sites compared with the magnetic carbon materials before sulfonation. The L acid sites were obviously more abundant than the B acid sites in the magnetic carbon materials both before and after sulfonation. Among them, Fe_3_O_4_@C-S exhibited the highest amount of B acid. Fe_3_O_4_@C-H showed a moderate acid density and the ratio of B/L acid site density.

The pyridine infrared characterization indicates that the Fe_3_O_4_@C-S and Fe_3_O_4_@C-H catalysts possess significantly more B, L, and total acid sites than Fe_3_O_4_@C, implying better catalytic performance. The higher acid site density indicates that there are more acid sites on the catalyst surface for adsorption and conversion of reactants. During hydrolysis reactions, the magnetic carbon material swelled due to water interaction. The internal acid sites came into contact with the substrate and exerted their catalytic effect.

### 2.2. Catalytic Performance of a Carbon-Based Solid Acid Catalyst

The main components of straw are cellulose and hemicellulose, which are hydrolyzed to produce glucose and pentose, respectively, under the action of solid acid catalysis. To compare the catalytic activity of the catalyst by the two sulfonation methods, rice straw was selected as the hydrolysis substrate, and identical time gradients were set. The yield of reducing sugars from the hydrolysis of rice straw catalyzed by two sulfonated magnetic carbon microspheres were determined. The results are shown in Table 2. The highest glucose yields using Fe_3_O_4_@C-S and Fe_3_O_4_@C-H were 29.35% and 22.23%, which is relatively low, indicating that the performance of the two catalysts for hydrolyzing cellulose is limited. The hydrolysis efficiency of the concentrated sulfuric acid sulfonation catalyst for cellulose was better than that of hydroxyethyl sulfonic acid sulfonation catalyst, which may be attributed to the higher ratio of B/L acid density on the surface of Fe_3_O_4_@C-S promoting the hydrolysis of cellulose. Fe_3_O_4_@C-H led to a peak pentose yield of 76.67% in 110 min, which was slightly higher than that of 76.51% in 90 min in the presence of Fe_3_O_4_@C-S. This phenomenon is due to the fact that the lower ratio of B/L acid density on the surface of Fe_3_O_4_@C-H is conducive to the hydrolysis of hemicellulose. The results indicated that the catalytic properties of Fe_3_O_4_@C-H and Fe_3_O_4_@C-S in promoting the hydrolysis of hemicellulose into pentose are similar. This suggests that the hydroxyethyl sulfonic acid could be substitute for sulfonation with concentrated sulfuric acid to prepare a carbon-based solid acid with -SO_3_H, which was used in upgradation of biomass to reducing sugar. As a green sulfonating agent, the hydroxyethyl sulfonic acid could be applied to replace the concentrated sulfuric acid, which would mitigate the environmental pollution. Compared with the previous research on sulfonated carbon catalysis, the preparation method of the catalyst has been innovated in this paper. The sulfonation degree and stability of the catalyst were effectively improved by adopting a more environmentally friendly sulfonation method. Therefore, research on catalysts sulfonated with hydroxyethyl sulfonic acid is significantly important and valuable. During the sulfonation process, the hydroxyl group in hydroxyethyl sulfonic acid could introduce sulfonic groups into the magnetic carbon material. This allows for the incorporation of a sufficient number of acidic active sites, thereby achieving a sulfonation effect similar to that of concentrated sulfuric acid [48].

**Figure 4:**
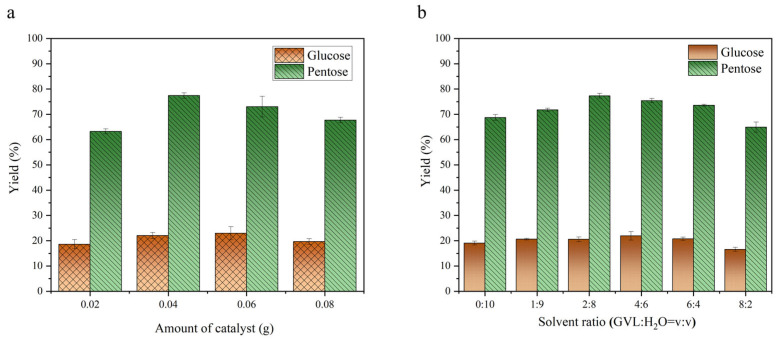
Effect of catalyst amount (**a**) and solvent ratio (**b**) on conversion of rice straw to glucose and pentose catalyzed by Fe_3_O_4_@C-H (reaction condition: 0.04 g rice straw, 140 °C, 110 min).

According to the above results, the catalyst for sulfonation of hydroxyethyl sulfonic acid was used as the catalyst object. The optimum experimental conditions for catalytic rice straw production of reducing sugar were studied. For multiple parameters (reaction time, temperature, solvent ratio, catalyst amount), we used single-factor experiments. The impact of catalyst dosage on the reaction is illustrated in Figure 4a. The magnetic carbon microsphere catalyst was sulfonated with hydroxyethyl sulfonic acid in the following amounts: 0.02 g, 0.04 g, 0.06 g, and 0.08 g. The rice straw was used as a substrate at 140 °C for 110 min. The highest yields of glucose and pentose sugars were obtained with catalyst dosages of 0.04 g. Increasing the amount of catalyst in the range of 0–0.04 g can significantly improve the yield, possibly because more active sites are provided. When the dosage is greater than 0.04 g, the decrease in yield may be caused by excessive catalyst, which reduces the selectivity of the product. However, since the hydrolysis of cellulose is generally more challenging than that of hemicellulose, the overall glucose yield was relatively lower. Therefore, considering all factors, a dosage of 0.04 g of the hydroxyethyl sulfonic acid sulfonated catalyst (Fe_3_O_4_@C-H) was deemed more effective for the hydrolysis experiments.

Suitable solvents and additives can improve the activity and selectivity of catalysts. In this study, water and gamma-valerolactone were used as solvents for the catalytic reaction. The effect of solvent ratio on the reaction outcome is illustrated in Figure 4b. A mixture of 0.04 g of the catalyst sulfonated with hydroxyethyl sulfonic acid and 0.04 g of rice straw was used after exploring the amount of catalyst. Then, these were combined with different gradients of GVL/H_2_O solvent systems. After reacting at 140 °C for 110 min, the yield of reducing sugars was compared across different solvent ratios. The results showed that the highest yield of reducing sugars from rice straw was observed with a GVL/H_2_O ratio of 2:8. The yields were higher than those obtained with pure water. GVL could reduce the conversion of xylose to furfural and condensation reaction of reducing sugars with intermediate molecules [49]. This indicated that by controlling the proportion of GVL in the solvent system, the yield of reducing sugars could be improved.

**Figure 5:**
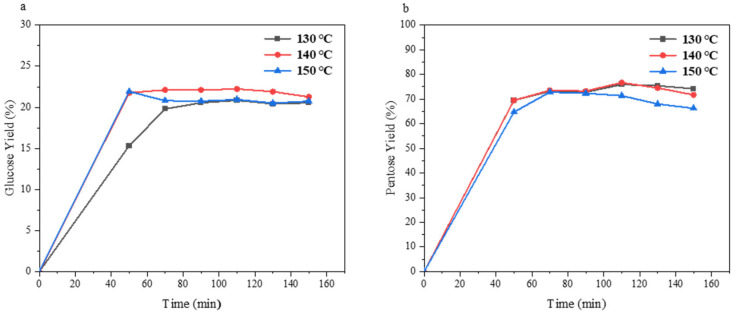
Effect of temperature and time on conversion of rice straw to glucose (**a**) and pentose (**b**) (reaction condition: 0.04 g rice straw, 0.04 g catalyst, 0.8 mL GVL, 3.2 mL distilled water).

In addition, the effects of reaction temperature and time on the production of reducing sugar were also investigated. The conversion of cellulose and hemicellulose in straw can be increased by increasing the temperature and prolonging the reaction time. The results of reaction temperature and reaction time on the catalytic reaction are illustrated in Figure 5. Hydrolysis reactions were conducted using 0.04 g of the catalyst with rice straw as substrates at temperatures of 130 °C, 140 °C, and 150 °C. Reaction times ranged from 50 to 150 min. At the same temperature, the experimental groups were set every 20 min to correspond to different reaction times. The highest yield of reducing sugars was achieved at 110 min under all three temperatures. Comparing the three reaction temperatures, the experimental groups treated at 140 °C showed higher yields of glucose as well as significant yields of xylose and arabinose [50]. The peak yields of glucose and pentose were 22.23% and 76.67%, respectively, which were obtained under 140 °C for 110 min. This may be due to a too long reaction time or too high of temperature resulting in by-products or catalyst deactivation.

**Figure 6:**
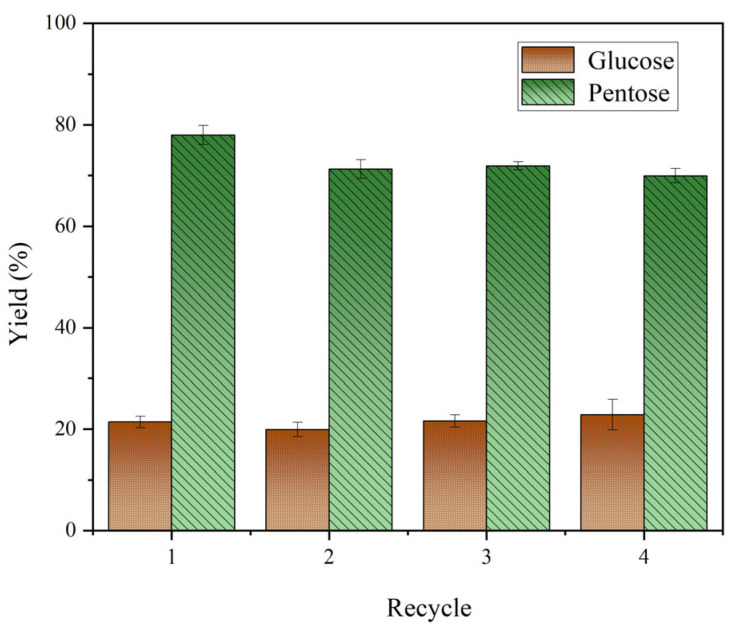
Recycling of Fe_3_O_4_@C-H for converting rice straw to glucose and pentose (reaction condition: 0.04 g rice straw, 0.04 g catalyst, 140 °C, 110 min, 0.8 mL GVL, 3.2 mL distilled water).

Due to the unique properties of magnetic carbon microspheres, the catalyst could be recovered after the reaction using a magnet. The recovered magnetic carbon microspheres were washed multiple times with water and alcohol to remove surface GVL until the solution reached a pH of approximately 7. The washed product was then dried overnight in an oven at 105 °C before being reused. The solid catalyst from the hydrolysis of rice straw at 140 °C for 110 min was recovered and reused under the same reaction conditions for multiple cycles [51,52]. As shown in Figure 6, the catalytic efficiency of the magnetic carbon microsphere catalyst slightly decreased after one reaction cycle. After four cycles, the yield of pentose sugars and glucose decreased by 5.71% and 1.51%, respectively. This phenomenon may be due to a decrease in the density of the acid sites in the catalyst or the inactivation of the structure. Some carbon deposits may have accumulated on the surface of the catalyst, causing the acid site to be covered and the acid site density to be reduced. Additionally, impurities or by-products in the reactants may interact with the acid site, rendering it inactive, reducing the catalytic efficiency. However, the specific catalyst deactivation factors still need to be further explored in the future, such as regeneration experiments. This is important for the lifetime and regeneration potential of the catalyst. This demonstrated that the magnetic carbon microsphere catalyst sulfonated with hydroxyethyl sulfonic acid has excellent stability and recyclability. However, further research on catalyst deactivation is still needed.

In summary, this study investigated the performance of two sulfonation catalysts for hydrolysis of rice straw to produce reducing sugars (glucose and pentose). The results show that the Fe_3_O_4_@C-H catalyst has similar catalytic performance to Fe_3_O_4_@C-S. Hydroxyethyl sulfonic acid has good biodegradability, which makes it not accumulate in the environment compared with traditional sulfonating agents, thus reducing the burden on the environment. Therefore, we further explored the optimum experimental conditions for the sulfonation catalyst. By comparing the amount of catalyst, ratio of reaction solvent, reaction time, and reaction temperature, the optimum conditions in the laboratory were obtained (0.04 g rice straw, 0.04 g catalyst, H_2_O/GVL ratio 8:2, 110 min at 140 °C). The pentose yield reached 76.67% and glucose yield reached 22.23%. In addition, the catalyst prepared in this study has magnetic properties that allow it to be easily separated by magnets after the reaction. The stability and catalytic performance of the catalyst remained high after four cycles of recycling.

## 3. Experimental Methods

### 3.1. Materials

FeCl_3_ (AR, 99%), Fe_3_O_4_, sodium polyacrylate, NaOH (95%), and citric acid trisodium salt (98%) were purchased from Shanghai Macklin Biochemical Co., Ltd. (Shanghai, China). FeCl_2_·4H_2_O was purchased from Tianjin ZhiYuan Reagent Co., Ltd. (Tianjin, China) Sulfuric acid (AR) was procured from Xihua, XiLONG SCIENTIFIC (Shantou, China). Hydroxyethanesulfonic acid (80%), ethanol (≥99.8%), and D-(+)-Xylose were purchased from Shanghai Aladdin Biochemical Technology Co., Ltd. (Shanghai, China). Ultra-pure water was used throughout in this work.

### 3.2. The Preparation of the Catalyst

#### 3.2.1. Preparation of Magnetic Nanoparticles Fe_3_O_4_

We dissolved FeCl_3_ and FeCl_2_·4H_2_O in distilled water under specific conditions, mixing thoroughly with a magnetic stirrer. We slowly added a 2 mol/L NaOH solution until the pH reached approximately 11, leading to the oxidation and hydrolysis of FeCl_3_ and FeCl_2_ and the formation of magnetic nanoparticles. We introduced a certain amount of trisodium citrate, then heated the solution to 80 °C and stirred continuously for 1 h. We separated the magnetic nanoparticles from the supernatant using a magnet and washed them repeatedly with ethanol and distilled water to remove impurity ions. We dried the precipitate in a vacuum drying oven at 60 °C, grinded it in a mortar after drying, and placed it into a centrifuge tube.

#### 3.2.2. Preparation of Fe_3_O_4_@C Composite

We mixed 0.4 g of Fe_3_O_4_ and 4.0 g of xylose in 60 mL of H_2_O at a mass ratio of Fe_3_O_4_ to xylose of 1:10, then added 0.05 g of sodium polyacrylate. We stirred the mixture on a magnetic stirrer for 30 min to ensure complete dispersion, then transferred it to a reaction vessel and carbonized at 180 °C for 24 h. After the reaction, we performed magnetic separation, washed sequentially with water and ethanol, and vacuum dried at 60 °C for 12 h to obtain the Fe_3_O_4_@C composite.

#### 3.2.3. Sulfonation of Fe_3_O_4_@C Composite

We combined 1.0 g of Fe_3_O_4_@C with 20 mL of concentrated sulfuric acid or 20 mL of hydroxyethyl sulfonic acid in a beaker, using concentrated sulfuric acid as a control. We stirred with a magnetic stirrer for 30 min to mix thoroughly, then transferred to a 100 mL polytetrafluoroethylene (PTFE)-lined hydrothermal high-pressure reactor and heated in an oven at 180 °C for 4 h. We filtered the resulting product, washed with water and ethanol until the pH reached 7, dried in an oven, grinded after drying, and stored in a dry environment. The catalyst sulfonated by concentrated sulfuric acid was named Fe_3_O_4_@C-SO_3_-S(Fe_3_O_4_@C-S), and the catalyst sulfonated by hydroxyethyl sulfonic acid was named Fe_3_O_4_@C-SO_3_-H(Fe_3_O_4_@C-H) [53].

### 3.3. Characterization and Analysis of Catalysts

The hydrothermally synthesized carbon materials, as well as the carbon-based solid acid materials sulfonated with concentrated sulfuric acid and hydroxyethyl sulfonic acid, were characterized using various analytical techniques. X-ray diffraction patterns were obtained using a BRUCKER D8 ADVANCE diffractometer (Bruker, Mannheim, Germany) with a copper target. The scanning angle range was 10–90°, and the scanning rate was 10°/min. The data analysis was performed using JADE 6.0 software. The morphological features of the three materials were examined using a ZEISS sigma 500 scanning electron microscope (ZEISS, Oberkochen, Germany). The elemental distribution of the three materials was observed using the thermal field emission scanning electron microscope JSM-7800F. The infrared spectra were recorded using a Nicolet IS10 FTIR spectrometer (Thermo Fisher Scientific, Waltham, MA, USA). The spectral range was 400–4000 cm^−1^ with a resolution greater than 0.4 cm^−1^. Desorption measurements of the three materials were performed using the Nicolet 380 spectrometer (Thermo Fisher Scientific, Waltham, MA, USA) at 150 °C and 200 °C, collecting the infrared spectrum of adsorptive pyridine in the range of 650–4000 cm^−1^. The specific surface area and porosity of all materials were measured using a BSD-PS1 surface area analyzer (Beijing Beishide Instrument Technology Co., Ltd., Beijing, China). N_2_ physical adsorption was employed, and the materials were degassed at 120 °C for 7 h. The pore size distribution and total pore volume were determined based on the BJH method applied to the adsorption isotherm data.

### 3.4. Test of Catalytic Performance of Catalyst for Production of Reducing Sugar

The composition of rice straw was determined using the two-step hydrolysis method developed by the National Renewable Energy Laboratory (NREL) in the USA [54]. The hydrolysate was analyzed by high-performance liquid chromatography (HPLC) to determine the concentrations of glucose, xylose, and arabinose. The cellulose and hemicellulose contents were calculated based on the measured sugar concentrations. The mass of ash and lignin was determined from the solid residue, and the ash content was further quantified by calcination, allowing the determination of lignin content. Rice straw is composed of 36% cellulose, 15.5% hemicellulose, 3.5% lignin, 9.8% ash, and 35.1% other components.

A mixture of 0.04 g of rice straw and a synthesized catalyst in a specific ratio of H_2_O/GVL was shaken well and placed in a 15 mL thick glass tube. Various sets of single-factor experiments were conducted by adjusting the reaction temperature, time, catalyst amount, and solvent ratio. The reaction mixture was filtered through a 0.22 μm PVDF membrane and analyzed by liquid chromatography. The remaining solid was dried in an oven at 60 °C, and a magnet was used to separate the catalyst from the reaction residue for catalyst reuse. The optimal reaction conditions were determined by selecting the appropriate catalyst and adjusting the catalyst amount, hydrolysis temperature, and reaction time based on the measured results.

The concentrations of glucose, xylose, and arabinose in the liquid samples were determined using an Agilent HPLC system (Agilent Technologies, Santa Clara, CA, USA). The conditions for the HPLC analysis were as follows: the mobile phase was a 5 mmol/L H_2_SO_4_ solution with a flow rate of 0.6 mL/min, the column temperature was set at 35 °C, and a refractive index detector (RID) was used. The concentrations of the products were calculated based on the peak area and reference standards.

(1) $$Glucose\;Yield\left(\%\right)=\frac{Mass\;of\;glucose}{Mass\;of\;cellulose}\times 100\%$$

(2) $$Pentose\;Yield\left(\%\right)=\frac{Mass\;of\;pentose}{Mass\;of\;hemicellulose}\times 100\%$$

## 4. Conclusions

In this study, we successfully synthesized two carbon microsphere catalysts with magnetic recyclable properties by using two different sulfonating agents, concentrated sulfuric acid and hydroxyethyl sulfonic acid. The sulfonation catalyst using hydroxyethyl sulfonic acid (Fe_3_O_4_@C-H) showed obvious advantages over the sulfonation catalyst using concentrated sulfuric acid (Fe_3_O_4_@C-S) in the production of pentose from rice straw. Hydroxyethyl sulfonic acid was used as a green sulfonating agent, and its advantages compared with concentrated sulfuric acid were reflected in two aspects. 1. Hydroxyethyl sulfonic acid is mildly acidic and does not release volatile toxic gases during the catalytic process, reducing the production of harmful substances from the source. 2. Its unique water solubility and biodegradability mean the waste liquid after the reaction can be directly treated by simple dilution, avoiding the complicated waste liquid neutralization step in the traditional sulfuric acid process, and significantly reducing the risk of secondary pollution. Through optimization, we determined that under the best experimental conditions (0.04 g rice straw, 0.04 g Fe_3_O_4_@C-H catalyst, H_2_O/GVL ratio 8:2, 110 min at 140 °C), the pentose yield reached 76.67% and the glucose yield was 22.23%.

Compared with other studies, the catalyst synthesized in this study combines the advantages of magnetic and sulfonation treatment and improves the recovery and catalytic performance of the catalyst. The reducing sugar yield we obtained under optimized conditions increased by 5–10% compared to previous studies [55]. In addition, the stability of the catalyst also showed obvious advantages. Compared with the activity decrease of 10–15% reported in the literature [29], the activity of the catalyst in this study only decreased by 5% after the fourth cycle. Nevertheless, there are still some unexplored aspects of this study. The prepared catalysts resulted in lower glucose yields, which limits their use as catalysts for glucose production. Although the two catalysts are similar in catalytic performance, performance differences may still lead to limited choice of catalysts in specific application scenarios. We look forward to further improving the synthesis method and structure of the catalyst in the future, which is expected to increase the output of glucose and pentose simultaneously. At the same time, the in-depth study of kinetics and reaction pathways is also very important, which is the key evidence for the comprehensive analysis of the transformation process of biomass and the design of catalysts. In addition to the rice straw used in this paper, other raw biomass materials as catalyst substrates were explored. We explored the industrial application of catalysts and provide effective strategies for the high-value utilization of bio mass.

## Figures and Tables

**Table 1:** The acid sites’ density of Fe_3_O_4_@C, Fe_3_O_4_@C-S, and Fe_3_O_4_@C-H.

Sample	Temperature (°C)	Number of Acid Sites (μmol/g)			B/L
		B Acid	L Acid	Total Acid	
Fe_3_O_4_@C	150	2.374	113.175	115.550	0.021
	200	1.770	29.117	30.887	0.061
Fe_3_O_4_@C-S	150	15.164	122.268	137.432	0.124
	200	13.210	48.156	61.366	0.274
Fe_3_O_4_@C-H	150	10.906	188.157	199.063	0.058
	200	6.397	93.819	100.217	0.068

**Table 2:** The yield of glucose and pentose catalyzed by Fe_3_O_4_@C-S and Fe_3_O_4_@C-H.

Time (min)	Glucose Yield (%)		Pentose Yield (%)	
	Fe_3_O_4_@C-S	Fe_3_O_4_@C-H	Fe_3_O_4_@C-S	Fe_3_O_4_@C-H
50	29.35	21.76	73.15	69.49
70	28.58	22.12	74.56	73.55
90	28.91	22.11	76.51	73.25
110	28.36	22.23	74.25	76.67
130	26.61	21.91	71.79	74.54
150	26.26	21.27	68.24	71.39

Reaction condition: 0.04 g rice straw, 0.04 g catalyst, 140 °C.

## Data Availability

The original contributions presented in this study are included in the article/Supplementary Materials. Further inquiries can be directed to the corresponding authors.

## Supplementary Materials

The following supporting information can be downloaded at: https://www.mdpi.com/article/10.3390/molecules30132675/s1, Figure S1: the magnetism of Fe_3_O_4_@C-H catalyst; Table S1: Textural properties of Fe_3_O_4_@C, Fe_3_O_4_@C-S and Fe_3_O_4_@C-H; Figure S2: Textural properties of Fe_3_O_4_@C, Fe_3_O_4_@C-S and Fe_3_O_4_@C-H; Figure S3: The EDX image of Fe_3_O_4_@C catalyst; Figure S4: The EDX image of Fe_3_O_4_@C-S catalyst; Figure S5: The EDX image of Fe_3_O_4_@C-H catalyst.
