# Supplementary material for: A Pathway for Sugar Production from Agricultural Waste Catalyzed by Sulfonated Magnetic Carbon Microspheres
Source: Molecules. 2025 Jun 20;30(13):2675. doi: 10.3390/molecules30132675 (PMC12251338; doi:10.3390/molecules30132675)
Supplement: Supplementary file 1 [file molecules-30-02675-s001.zip › molecules-3606160-supplementary.pdf]

Supplementary Materials

A Pathway for Sugar Production from Agricultural Waste Catalyzed by Sulfonated Magnetic Carbon Microspheres

Maoru Xu [1,†], Yanfeng Duan [2,†], Hongfu Li [1], Shoulin He [1], Xingyu Zi [1], Yanting Zhao [1], Cheng Jiao [1,*] and Xiaoyun Li [2,*]

1 Dali Tobacco Bureau of Yunnan Province, Dali 671000, China; xmaoru1856@163.com (M.X.); zy_huang2022@163.com (H.L.); he0325@163.com (S.H.); gkysciaccept@163.com (X.Z.); 18987237598@163.com (Y.Z.)

2 School of Agriculture and Biotechnology, Sun Yat-sen University, Shenzhen 518107, China; duanyf7@mail2.sysu.edu.cn

* Correspondence: jiaocheng2025@163.com (C.J.); lixy655@mail.sysu.edu.cn (X.L.)

† These authors contributed equally to this work.

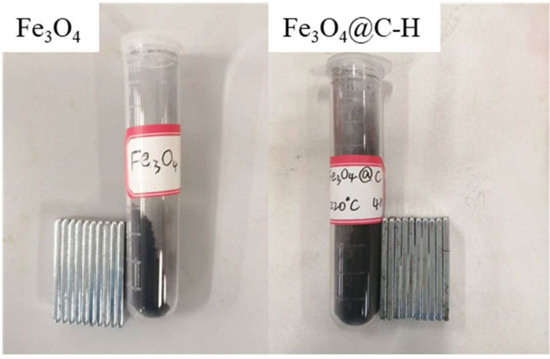

Figure S1 the magnetism of Fe_3O_4@C-H catalyst

Table S1 Textural properties of Fe_3O_4@C, Fe_3O_4@C-S and Fe_3O_4@C-H

Sample	S_{BET} (m^2/g)	V_p (cm^3/g)	D_a (nm)
Fe_3O_4@C	13.0379	0.0684	20.9850
Fe_3O_4@C-S	0.5893	0.0044	29.8650
Fe_3O_4@C-H	4.6113	0.0216	18.7365

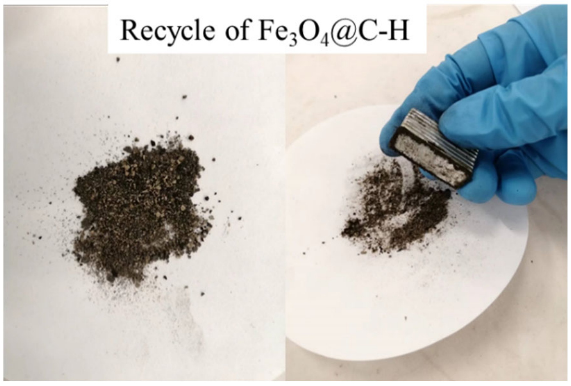

Figure S2 The Fe_3O_4@C-H catalyst is recovered by magnetic suction.

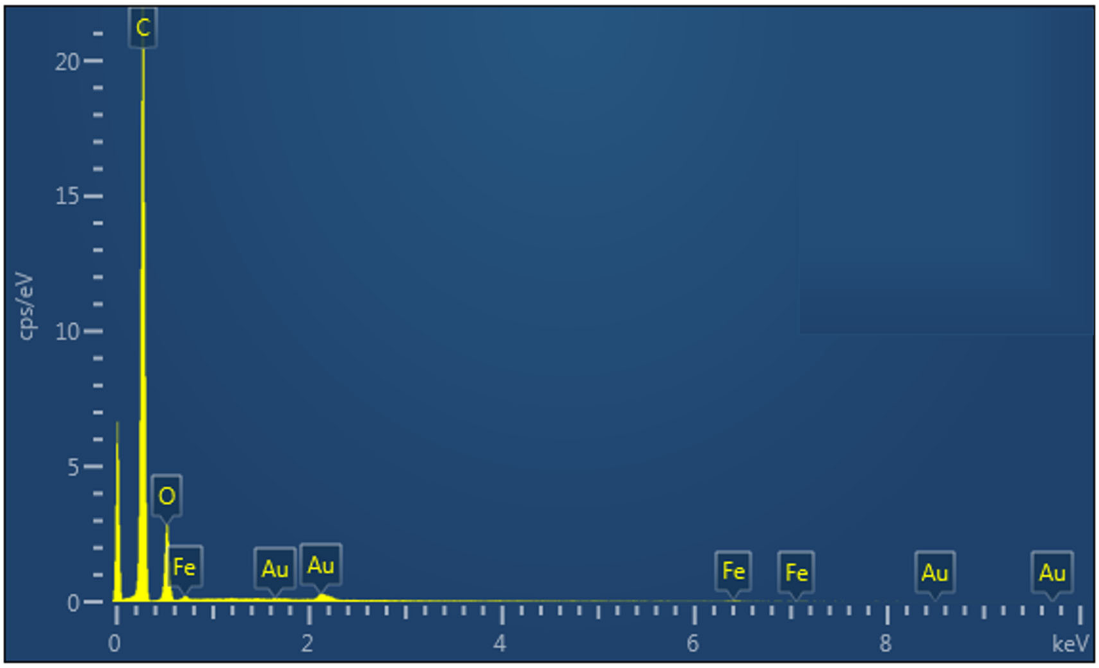

Figure S3 The EDX image of Fe_3O_4@C catalyst

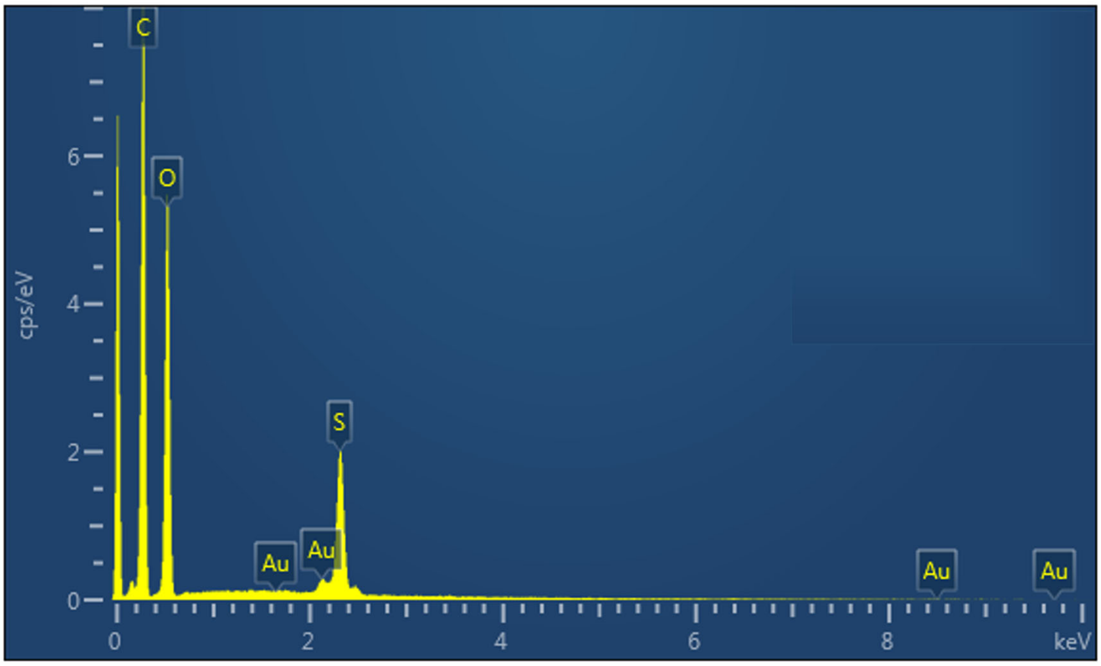

Figure S4 The EDX image of Fe_3O_4@C-S catalyst

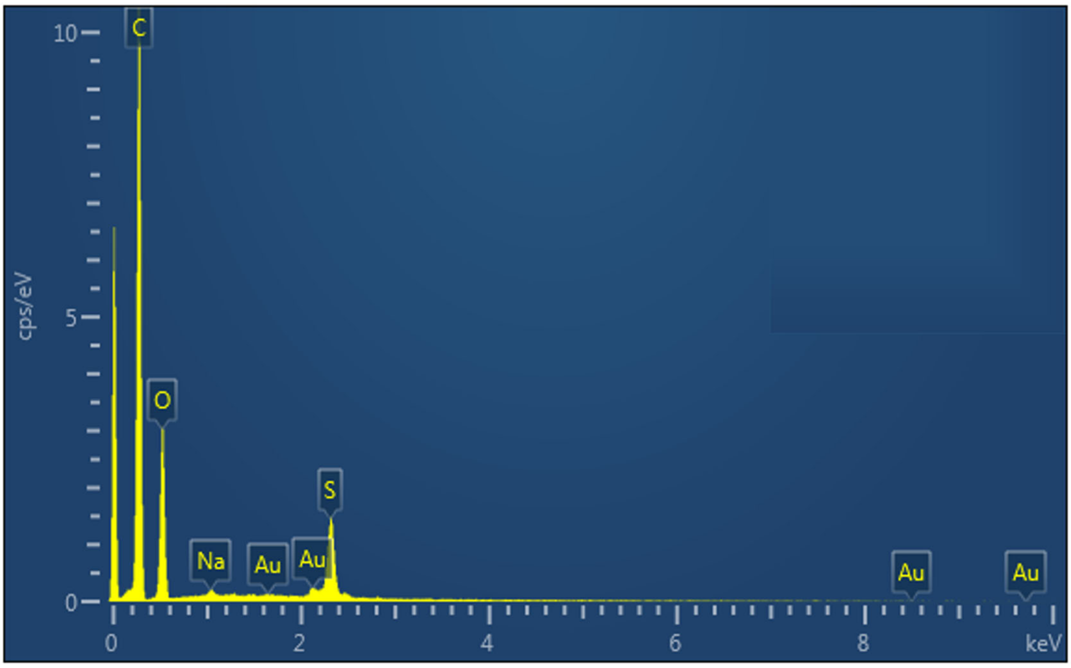

Figure S5 The EDX image of Fe_3O_4@C-H catalyst
